# Door – to – door immunization strategy for improving access and utilization of immunization Services in Hard-to-Reach Areas: a case of Migori County, Kenya

**DOI:** 10.1186/s12889-019-7415-8

**Published:** 2019-08-07

**Authors:** Duncan N. Shikuku, Maxwell Muganda, Soudie O. Amunga, Elly O. Obwanda, Alice Muga, Thomas Matete, Paul Kisia

**Affiliations:** 1 Afya Halisi Project, Migori County, Kenya; 2 Department of Health, Migori County, Kenya

**Keywords:** Immunization, Hard – to – reach, Immunization coverage, Access and utilization

## Abstract

**Background:**

Access to quality essential healthcare services and vaccines for all is key to achieving universal health coverage. Inequities driven by differences in place of residence and socio-economic status persist among different communities hindering the achievement of sustained performance on immunization indicators. Innovative community-based Reach Every Child (REC) interventions at the sub-county and county level can reduce these local inequities. This study determines the effect of an enhanced door-to-door immunization strategy on improving immunization coverage in hard-to-reach areas of Migori.

**Methods:**

This was a cross-sectional review of District Health Information System 2 immunization data for July and August 2018 for Migori County. During the presidential immunization rapid results initiative (RRI) in July 2018, poorly performing wards/facilities were mapped using the Quantum Geographic Information Systems methodology, and unreached rural-urban populations identified. Through review of facility level Kenya Expanded Programme on Immunization data, 64 health facilities with over 100 unimmunized children each between January 2017 and June 2018 in all sub-counties were prioritized. In August 2018, intensified fixed-point immunization services were offered within the prioritized facilities. Further, a 3-day door-to-door defaulter tracing by community health volunteers and household level immunization by nurses was conducted. Immunization coverage performance for access and utilization for the two periods were compared using z-tests/t-tests.

**Results:**

Cumulatively, a total of 10,744 and 14,809 children were reached with immunization in July and August respectively for the 64 facilities. There were significant increases in the immunization coverage for BCG (74.4% vs 89.9%, *P* = 0.0001), Penta 1(96.2% vs 102%, *P* = 0.0649), Penta 3 (92.3% vs 112.1%, *P* = 0.0001), MR1 (81.7% vs 111.5%, *P* < 0.0001) and the fully immunized children at 1 year (78.6% vs 103.9%, *P* < 0.0001). Penta 3 and MR1 drop-out rates (3.99% vs − 9.86%, *P* = 0.0007; 15.06% vs − 9.27%, *P* = 0.0001 respectively) decreased significantly. Similar significant effects were observed at the subcounty levels (*P* < 0.05).

**Conclusion:**

Hard-to-reach populations require multiple REC strategies to reach every child with immunization. Health facilities should actively analyze and use routine immunization data and invest in community health strengthening systems to identify hard-to-reach areas to be targeted with outreaches to improve immunization coverage.

## Background

Immunization saves an estimated 2–3 million lives every year [1] and is amongst the most cost – effective public health interventions for reducing global child morbidity and mortality [2, 3]. In Sub-Saharan Africa, despite the availability of vaccines against many infectious diseases and efforts from governments and their partners to vaccinate every child, the mortality rate from vaccine – preventable diseases for children under-five remains among the highest in the world [4].

Globally, Diphtheria-Tetanus-Pertussis (DTP3) coverage remains at 85% in 2017, leaving 19.9 million children vulnerable to vaccine preventable diseases. The 2018 Global Vaccine Action Plan (GVAP) report and other evidence indicates that continuing mass urbanization and migration, population growth, geographical and socio – economic inequities, place of residence and environmental disruptions in the communities continue to present major challenges to national immunization systems [1, 5–13] especially among the most disadvantaged, marginalized and hard-to-reach populations in both rural and urban settings. Globally, it is estimated that 85% of children have been vaccinated with the first dose of measles vaccine by the age of 1 year. This is below the World Health Organization (WHO) recommended measles immunization coverage of at the minimum 95%. Existence of pockets of low coverage in countries predisposes the region to large measles outbreaks causing many deaths [14]. For instance, in 2017, measles outbreaks were reported in three drought – affected countries in the Horn of Africa, including Ethiopia (3481 cases), Kenya (11 cases) and Somalia (7031 cases) [15]. The continued detection of circulating vaccine-derived poliovirus and the resurgence of measles outbreaks is further evidence that national immunization programs are not achieving the goal of reaching every child [1].

There have been significant improvements in the performance of the Expanded Program on Immunization (EPI) in Africa since its inception in 1974. However, there exist wide inter- and intra-country differences. While 35 (67%) countries reported 80% national DTP3 coverage in 2010, only 16 (30%) countries reported at least 80% DTP3 coverage in 80% of their districts [10]. Many structural, financial, geographical and managerial barriers to providing universal access to immunization exist in the region. Despite these obstacles, the region has achieved coverage levels of about 72% for three doses of the diphtheria-tetanus-pertussis (DTP3) vaccine and the first dose of the measles vaccine.

Various strategies have been developed globally to guide implementation of EPI services and improve coverage. The Reaching Every Community/Child (REC) strategy is an innovative “bottoms” up approach that seeks to improve immunization coverage at health facilities. It has five key operational elements: re – establishing outreach vaccination services, supportive supervision to healthcare workers, linking services with communities, monitoring and use of immunization data for action and planning and management of resources through microplans [16]. Operationalization of the REC approach has contributed to increasing DPT3 coverage in Africa from 57 to 80% between 2000 and 2014. However, fewer than 50% of African countries achieved the GVAP national targets of 90% DPT3 coverage by 2015 (16 countries), among which 13 maintained this level for 3 consecutive years. To maximize the full potential of vaccination, the GVAP [17] and the regional goals for Africa must target to achieve at least 90% vaccination coverage for routinely recommended vaccines at the national level and at least 80% in all the health districts/counties by 2020 [18]. Reviewing best practices and emerging issues as well as intensifying focus on closing the gaps in immunization services at the community level using innovative strategies can reduce the pockets of local inequities in REC. This includes more emphasis on community-based interventions at the sub-district level emphasizing on reducing inequity in immunization coverage, integration of health services, delivering vaccines beyond infancy using a life course approach, focusing on urban, poor and marginalized populations, and paying special attention to insecure and conflict areas in Africa [16].

Kenya’s fully immunization coverage rate as per the Kenya Demographic and Health Survey (KDHS) 2014 was 68% with stark differentials in immunization coverage existing across counties [19]. In 2017, only six of the 47 (13%) counties had DPT3 coverage of at least 90% and a paltry four counties (9 %) had Measles/Rubella 1 coverage of at least 95% – the lowest coverage reported in the country since 2011 [20]. This was contributed by the protracted industrial action by healthcare workers for most periods of the year that paralyzed operations in the public health facilities [21, 22]. Other challenges affecting immunization in Kenya include the scheduling immunization hence missed opportunities, knowledge gap in immunization, inadequate capacity for storage of vaccines, myths and misconceptions around immunization by the community and economic barriers (faith – based organizations and private sector charging immunization services). This does not resonate with the country’s national vaccines and immunization objectives of ensuring: equitable access to appropriate vaccination services for all persons; universal immunization of children with appropriate doses of Ministry of Health prescribed childhood vaccines; universal immunization of special risk groups with Ministry of Health approved priority vaccines; and optimum vaccination service delivery in response to specific situations of outbreak of life threatening vaccine – preventable diseases [23].

National county – level statistics show that Migori county had a low proportion of children 12–23 months (segregation at county level at 1 year not available) fully immunized at just 37% in 2014 [24]. This included BCG, measles, three doses each of DTP and polio vaccine (excluding polio vaccine given at birth) and pneumococcal. The 2016 service statistics paint a picture of inequalities existing in the county. For instance, Kuria West and Suna East subcounties have fully immunized children (FIC) rates of below 70% and drop – out rates of above 10% between the first and third doses of Pentavalent vaccine. Out of the total 662,004 unvaccinated children nationally between Jan 2017 and May 2018, Migori had 16, 760 unvaccinated children [25]. This was 36% of the county’s expected under 1 population for the year 2018 [26]. This paper determines the effect of an enhanced door-to-door immunization strategy on the immunization coverage in rural, urban and socio – economic hard-to-reach areas of Migori County, Kenya following the national immunization rapid results initiative (RRI).

## Methods

### Setting

**Fig. 1:**
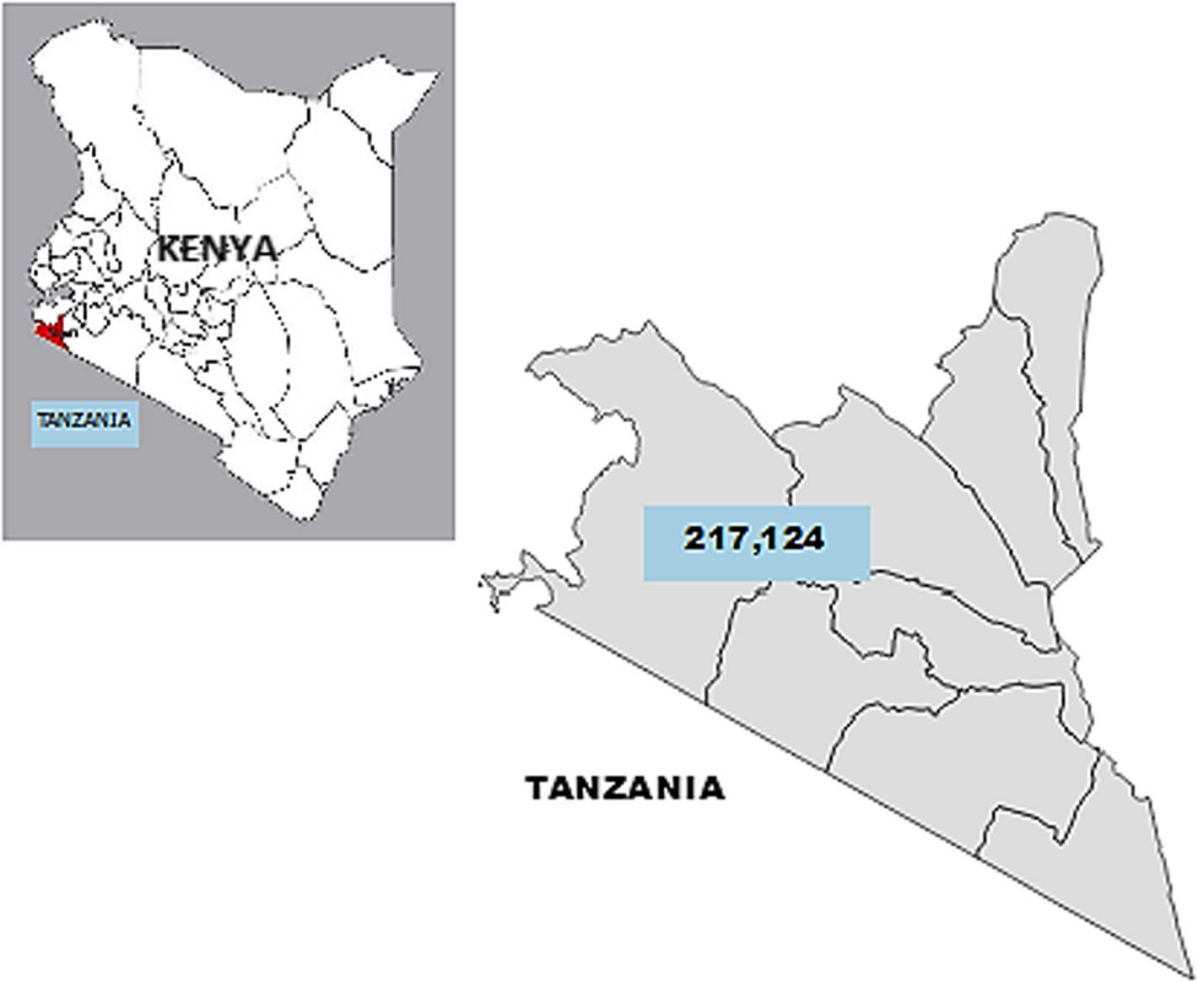
Map of Migori County showing the children under 5 years population in 2018. Authors’ own; generated using QGIS software

Migori County is found to the southwestern part of Kenya and borders Homa Bay County (North), Kisii County (North East), Narok (South East), Tanzania (West and South) and Lake Victoria to the West. The county also borders Uganda via Migingo islands in Lake Victoria. It is divided into eight subcounties. It has an area of 2586.4 km^2^ with an estimated population of 1,119,184 in 2018 [26]. It has a population density of 353 per square kilometer and 43% of the population live below the poverty line [27]. The main economic activities include agriculture, fishing, manufacturing and small – scale mining. The county has both rural and urban populations. Poor road network within the county limits access & utilization of health services especially during rainy seasons. The population around the Tanzania and Narok borders, fishing zones on the shores of Lake Victoria and the goldmines is migratory in nature depending on the existing cash flow and economic gains for daily living thereby playing disrupting continued uptake and utilization of health services including immunization. The county also has the Roho and Legio Maria religious sects that discourage use of health services (Fig. 1).

### Design

**Fig. 2:**
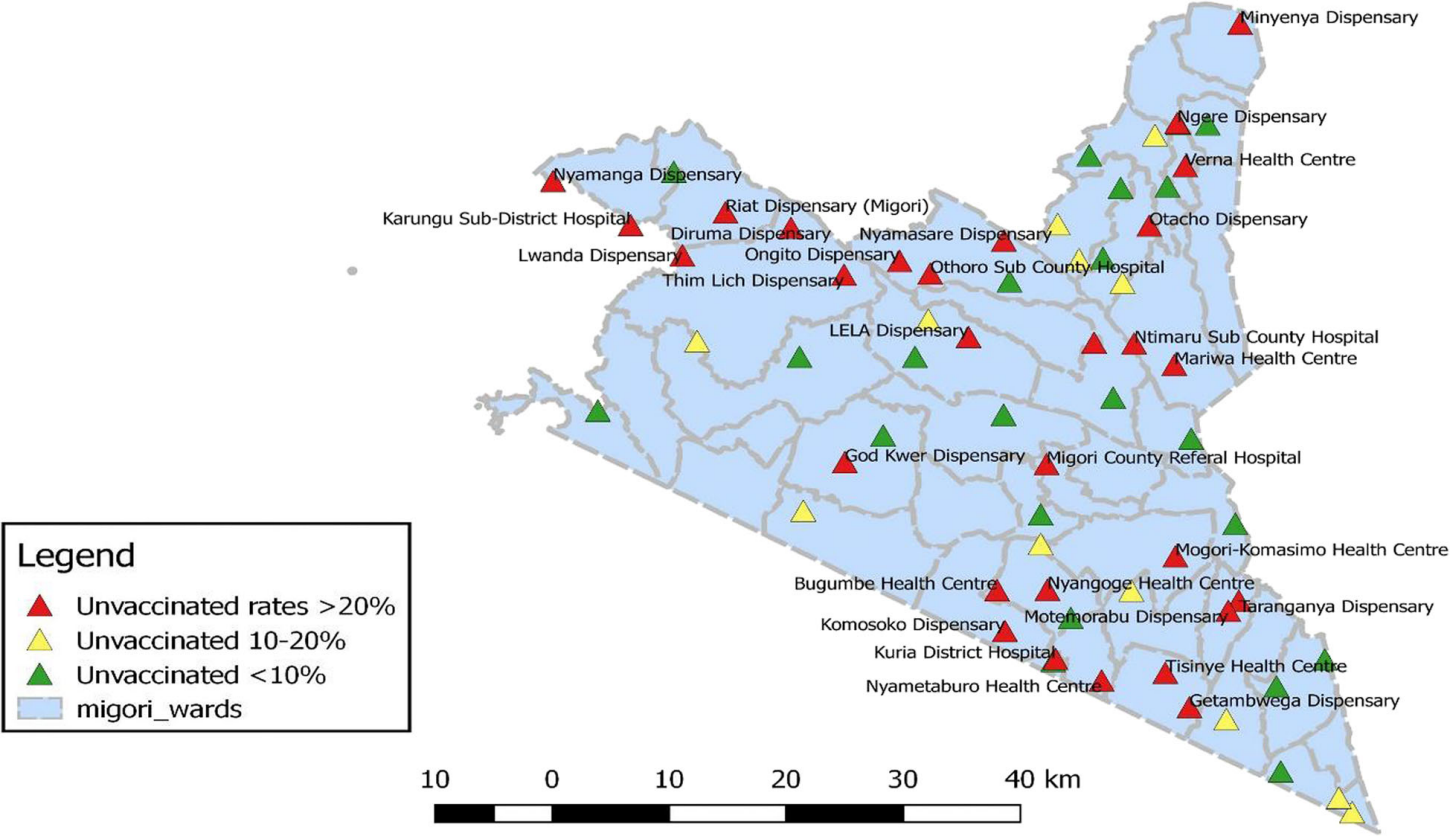
QGIS distribution of the 64 health facilities with the highest unvaccinated children in Migori County in July 2018. Authors’ own; generated using QGIS software. Door – to – door defaulter tracing, immunization and data collection

This was a cross – sectional review of District Health Information System 2 (DHIS2) immunization data for July and August 2018 for Migori County. During the presidential immunization RRI in July 2018, poorly performing wards/facilities were mapped using the Quantum Geographical Information System (QGIS) application that supports viewing, editing and analysis of geospatial data for the two study periods (Fig. 2). The unreached rural-urban and socio – economic hard –to – reach populations were identified. A total of 64 health facilities with over 100 unimmunized children each between January 2017 and June 2018 from all the eight subcounties were identified. In July, routine fixed-point immunization services were provided whereas in August, fixed – point plus a 3 – day enhanced door – to – door defaulter tracing by community health volunteers (CHVs) and household immunization by nurses was conducted in the 64 health facilities (out of 185 immunizing facilities) with high burden of unimmunized children. Each health facility was allocated three CHVs under the supervision of the community health assistant (CHA) and one frontline healthcare worker who was the vaccinator. The exercise was supervised by the subcounties’ health management teams (subcounty EPI coordinator and subcounty community strategy focal person). Overall supervision and coordination of logistics was provided by the county EPI logistics team. Importantly, the county, subcounties and the health facilities provide daily immunization services and had adequate stocks of routine vaccines both before and during the mobile immunization period. Afya Halisi, a 5-year USAID funded project facilitated the ground logistics – transportation, lunches and coordination airtime for the county and subcounty teams and lunches and transportation for the healthcare workers and the CHVs during the mobile exercise.

The select facilities generated the immunization defaulters lists from the Immunization Permanent Registers (MOH 510) and the immunization diaries. These lists were assigned to the CHVs from the catchment areas who mapped out the community based on the unvaccinated children’s burden. Community mobilization for the immunization services was conducted by the respective CHVs who provided households with information on benefits of starting and completing all the immunization services as per the prescribed MOH schedule. Together with the vaccinator, the team moved from house to house, scrutinized Mother Child Health (MCH) records (MOH 216) or the immunization card, BCG scar mark as well as mother’s or guardian’s verbal verification to identify the unimmunized children. The CHVs had earlier received basic orientation on the MCH records. Importantly, in areas considered vast, the team of CHVs mobilized all the eligible unvaccinated children to central points where the vaccinator would visit together with the CHA and vaccinate the children. To maintain the cold – chain of the antigens, the vaccinators carried the vaccines in cold boxes and vaccine carriers each day. After vaccination, the vaccinator and the CHA would tally the immunization tally sheet (MOH 702), update the MCH records, counsel the client on the importance of scheduled immunizations and book for the next appointment for the subsequent follow – ups/immunizations. The CHVs and the vaccinators updated their immunization defaulters’ records for reconciling/updating of the immunization permanent registers daily during the exercise. At the end of the exercise, the vaccinators updated the daily immunization data in the Immunization Services Uptake Summary (MOH 710) both the static/fixed point immunization services at the facility and the mobile immunization services. Data from 64 health facilities in Migori County were summarized and routinely entered in the national District Health Information System (DHIS2) by the subcounty health records officers. Technical assistance was provided to the health records team to ensure timely, complete, and accurate reporting of immunization indicators.

### Variables and measurements

Immunization coverage as per the immunization schedule at birth – 2 weeks, 6, 10 & 14 weeks, 9 months and at 18 months were computed as follows: birth – 2 weeks, BCG and OPV 0; 6 weeks – Pentavalent 1; 10 weeks – Pentavalent 2; 14 weeks – Pentavalent 3; 9 months – Measles/Rubella 1; proportion of fully immunized children at 1 year and at 18 months – Measles/Rubella 2. Drop – out rates for Pentavalent 3 and Measles 1 were also computed for utilization of immunization services. It is assumed that with adequate stocks for all antigens, each child receives all the due antigens at a given immunization visit as per the national immunization schedule.

### Data analysis

Immunization data for July and August 2018 were extracted from the DHIS2 and entered in Microsoft Office Excel 2013 for data cleaning. Cleaned data was then exported to STATA version 12 for analysis. Differences in proportions of the coverages and drop – outs for the two time periods were compared through z – tests (for the 64 facilities)/paired t – tests (for the 8 subcounties) and MOH summary data and program reports were also reviewed to triangulate the information obtained from the DHIS2. Drop-out rates were calculated as follows: (i) Penta1 to Penta3 dropout rate = (Penta1 – Penta3) ÷ Penta1 × 100%, where: Penta1 is the number (or percentage) receiving the first pentavalent vaccine dose and Penta 3 is the number (or percentage) receiving the third dose; (ii) Penta1 to Measles dropout rate = (Penta1 – Measles) ÷ Penta1 × 100%, where: Penta1 is the number (or percentage) receiving the first pentavalent vaccine dose and Measles is the number (or percentage) receiving the Measles dose [28]. Our study was not only interested in the significance test alone. The use of the parametric tests allowed us to acknowledge the special population from which the samples came, and this is best done with estimates of parameters and confidence intervals [29]. Confidence intervals were calculated at the 95% level and P – values ≤0.05 were considered statistically significant.

## Results

### Number of children immunized by age per key antigen

**Fig. 3:**
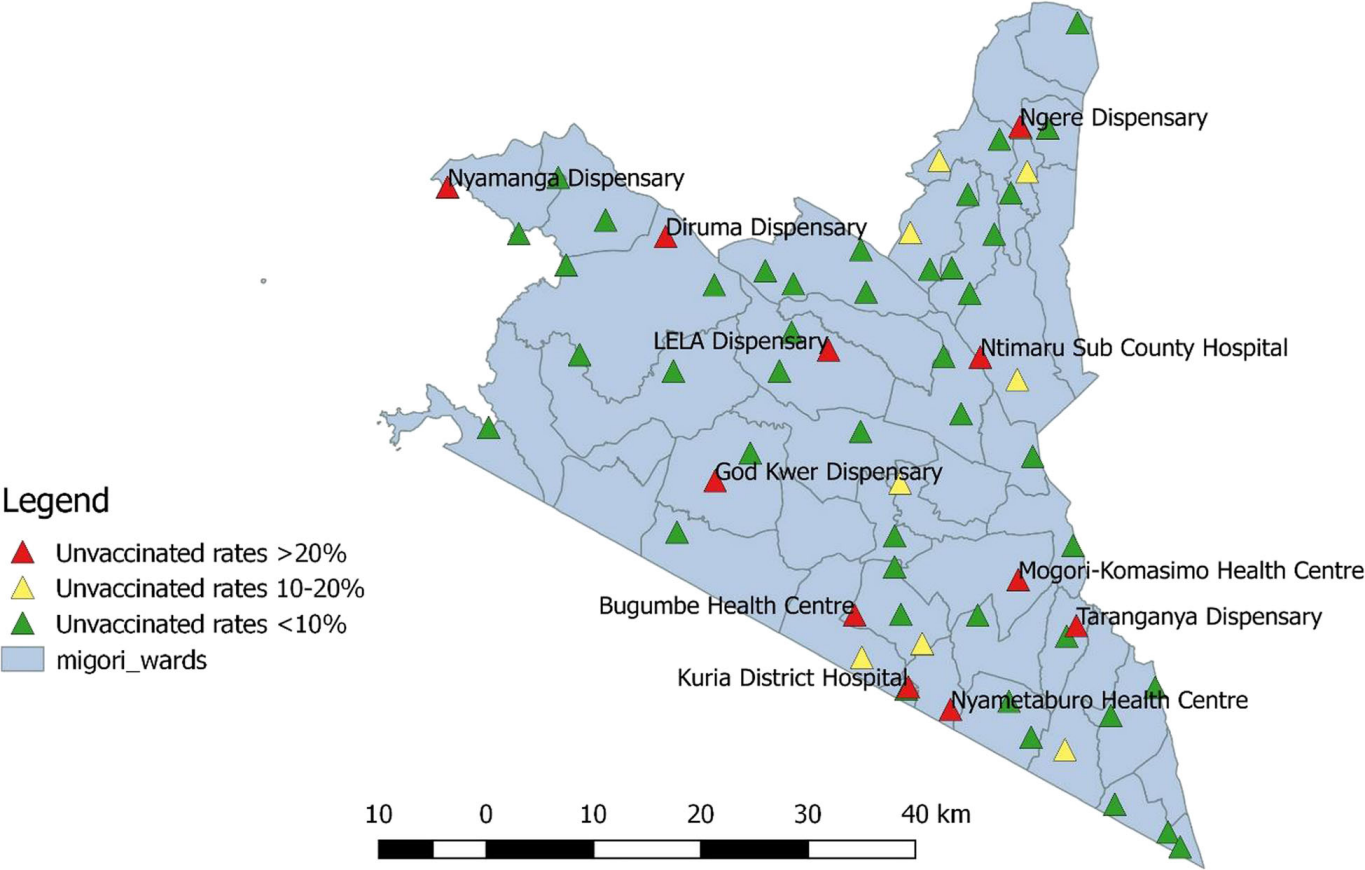
QGIS distribution of the unvaccinated children in the 64 facilities after the mobile immunization strategy in Migori County in August 2018. Authors’ own; generated using QGIS software

Overall, there was a marked improvement in the coverage by facilities in the number of unimmunized children compared to the previous routine fixed immunization service delivery (Fig. 3).

The findings indicate an increase in the total number of children immunized per antigen and by age from July to August (facilities: 10744 vs 14,809; subcounties: 20543 vs 25,550) as indicated in Table 2 below. At the facility level, the increase was more marked in children reached over the age of 1 year for BCG (19 times), Pentavalent 1 (22 times), Pentavalent 3 (19 times), MR1 (5 times) and MR2 (9 times) antigens as per the recommended immunization schedule. About 7 % (296 of 4396) of the children who received MR1 were above the age of 1 year. Similarly, about 30% (1211 of 4079) of the children received MR2 beyond 2 years of age. At the subcounty level, similar trends were observed as follows: Pentavalent 1 > 1 year (22 times), Pentavalent 3 > 1 year (11 times) and MR2 > 2 years (6 times) (Table 1).

**Table 1:** Total number of children immunized for July and August 2018 per antigen

	Total immunized (64 facilities)			Total immunized (8 subcounties)		
	July 2018	Aug 2018	% increase	July 2018	Aug 2018	% increase
OPV 0	2053	2205	7.4	3337	3719	11.4
BCG < 1 yr	2016	2301	14.1	3562	4019	12.8
BCG > 1 yr	1	19	1800.0	9	25	177.8
PENTA 1 < 1 yr	2068	2163	4.6	3863	3999	3.5
PENTA 1 > 1 yr	1	22	2100.0	1	22	2100.0
PENTA 2 < 1 yr	1874	2225	18.7	3627	4053	11.7
PENTA 2 > 1 yr	4	30	650.0	7	35	400.0
PENTA 3 < 1 yr	1952	2324	19.1	3711	4242	14.3
PENTA 3 > 1 yr	4	74	1750.0	7	77	1000.0
MR1 < 1 yr	1738	2362	35.9	3547	4326	22.0
MR1 > 1 yr	53	243	358.5	108	331	206.5
MR2 < 2 yrs	907	1961	116.2	1888	3088	63.6
MR2 > 2 yrs	126	1085	761.1	213	1333	525.8
TOTAL	10,744	14,809	37.8	20,543	25,550	24.4
FIC at 1 yr	1671	2230	33.5	3412	4120	20.8

*BCG* Bacille Calmette Guerin, *PENTA* Pentavalent (Diphtheria-Tetanus-Pertussis-Hepatitis B-Hemophilus influenza type b), *MR* Measles/Rubella and *FIC* Fully Immunized Child

### Facilities immunization coverage and drop – outs for July and august 2018

Vaccination coverage by antigen for the facilities indicated that OPV 0 and BCG had lower coverages (less than 90%) of the expected per antigen for the two reference periods (OPV 0: 75.67 vs 84.76 and BCG: 74.44 vs 89.94). Vaccine coverages significantly increased from July to August for OPV 0 (75.6–84.8%, *P* = 0.0008, CI = 3.57–14.43), BCG (74.4–89.9%, *P* = 0.0001, CI = 8.01–14.98), Pentavalent 3 (92.4–112.0%, *P* = 0.0001, CI = 9.96–29.52) and Measles/Rubella 1 (81.7–111.5%, *P* < 0.0001, CI = 19.97–39.61). The proportion of children fully immunized by 1 year of age increased significantly from 78.56 to 103.86 (*P* < 0.0001, CI = 16.09–34.5). Drop – out rates for Penta 3 and Measles/Rubella 1 decreased significantly (*P* < 0.05) (Table 2).

**Table 2:** Two – point comparison of immunization coverage and drop – out per antigen for the 64 health facilities for the period of July and August 2018

OPV0 coverage	July	Aug	diff	P - value			95%CI
	75.67	84.76	9	0.0008*		3.57	14.43
BCG coverage	74.44	89.94	15.5	0.0001*		8.01	14.98
PENTA 1 coverage	96.19	102.03	5.84	0.0649		1.76	13.44
PENTA 3 coverage	92.35	112.09	19.74	0.0001*		9.96	29.52
Proportion of under 1 vaccinated against MR1	81.7	111.49	29.79	< 0.0001*		19.97	39.61
Proportion of under 1 fully vaccinated (FIC)	78.56	103.86	25.3	< 0.0001*		16.09	34.5
PENTA 3 drop - out rate	3.99	−9.86	−13.85	0.0007*		−23.32	−4.5
MR1 drop - out rate	15.06	−9.27	−24.34	0.0001*		−38.74	−11.38

*OPV* Oral Polio Vaccine, *BCG* Bacille Calmette Guerin, *PENTA* Pentavalent (Diphtheria-Tetanus-Pertussis-Hepatitis B-Hemophilus influenza type b), *MR* Measles/Rubella and *FIC* Fully Immunized Child

*P ≤ 0.05 statistically significant

### Subcounties immunization coverage and drop – outs for July and august 2018

The 64 facilities with the high numbers of unimmunized children had a net effect on the overall vaccination coverage and drop – outs per antigen at the subcounties’ level. All the vaccine coverages for OPV 0, BCG, Pentavalent 1, Pentavalent 3 and Measles/Rubella 1 were above 90% in August with the highest gains observed in Pentavalent 3 and Measles/Rubella 1. Drop – outs in Pentavalent 3 and Measles/Rubella 1 were all less than 10% (Pentavalent 3 drop – out: 3.97 vs − 5.28, *P* = 0.0084, CI = − 17.00 - -2.3) and MR1 drop - out: 8.02 vs − 7.82, *P* = 0.0006, CI = − 25.52 - -4.75) (Table 3).

**Table 3:** Two – point comparison of immunization coverage and drop – outs per antigen for the 8 subcounties

OPV0 coverage	July	Aug	diff	P - value	95%CI		
	84.79	94.3	9.5	0.0024*		3.99	15.03
BCG coverage	86.91	97.71	10.8	0.0018*		4.84	16.74
PENTA 1 coverage	97.53	101.19	3.66	0.1773		5.08	12.4
PENTA 3 coverage	93.66	106.53	12.86	0.0021*		5.57	20.15
Proportion of under 1 vaccinated against MR1	89.71	109.1	19.39	0.001*		9.73	29.04
Proportion of under 1 fully vaccinated (FIC)	85.98	103.85	17.86	0.0013		8.64	27.11
PENTA 3 drop - out rate	3.97	−5.28	−9.25	0.0084*		−17.00	−2.3
MR1 drop - out rate	8.02	−7.82	−15.84	0.0006*		−25.52	−4.75

*OPV* Oral Polio Vaccine, *BCG* Bacille Calmette Guerin, *PENTA* Pentavalent (Diphtheria-Tetanus-Pertussis-Hepatitis B-Hemophilus influenza type b), *MR* Measles/Rubella and *FIC* Fully Immunized Child

*P ≤ 0.05 statistically significant

### County’s overall performance from Jan – sept 2018

Overall, the month of August registered the highest coverage with at least 90% coverage for all antigens and above 95% for MR1 as recommended (Table 4).

**Table 4:** County’s overall coverage from January–August 2018

Period	Jan	Feb	March	April	May	June	July	Aug
OPV 0 coverage	79.9	75.8	76.1	88.1	92.4	78.8	83.6	93.2
BCG Coverage	90.7	76.1	97.4	91.9	95.9	83.9	85.4	96.4
PENTA 1 coverage	92.9	94.5	88.3	91.8	98.3	95.4	96.8	100.2
PENTA 2 coverage	88.5	92.5	86.7	84.3	84.7	90.2	90.9	101.5
PENTA 3 coverage	106.9	96.2	89.9	92.1	88.8	85.7	93	106.5
MR 1 coverage	86	91.5	82.5	97.3	93.2	91	88.8	108.4
PENTA 3 dropout rate	−15.1	−1.8	−1.8	−0.28	9.7	10.2	3.9	−6.3
MR dropout rate	7.4	3.2	6.6	−6.0	−5.2	−4.6	8.3	−8.2

Source: DHIS2 accessed on 16th March 2019

## Discussion

The study was set to establish the effect of a door – to – door mobile immunization strategy on immunization access and utilization in hard – to – reach areas. Overall, the findings are suggestive that mobile immunization strategy improved access to and utilization of immunization services among those in hard – to – reach areas. With half of the subcounties bordering Tanzania, there is always the cross – border migration that occur throughout the year (plus ‘walk – in’ clients from the migratory population in the neighboring Narok County) owing to the porous borders that can also explain in some way the high coverage (over 100%) achieved during the mobile immunization services [30].

Vaccination coverage for birth doses (OPV 0 and BCG) and Measles/Rubella in the 64 facilities were below national target of 90% for doses recommended at 2 weeks of age for the two periods, indicating suboptimal access to immunization services. This finding is consistent with other studies conducted in urban and informal settings/slums [9, 13]. Despite progress in vaccine development and immunization delivery systems worldwide, populations in rural, urban and social – economic hard – to – reach settings often have limited or no access to lifesaving vaccines, leaving them at increased risk for morbidity and mortality related to vaccine – preventable disease. Evidence suggests that strengthening community mapping and monitoring of all pregnant women and the under 1 population by the community health strategy units can ensure that the population accesses skilled maternal, newborn and child health services [10, 31, 32]. This therefore calls for more targeted planning and combined approaches consonant with the Global Immunization Vision and Strategy (GIVS) of “using a combination of approaches to reach everyone targeted for immunization” [33] in these communities to track newborns for immunization with birth doses (upto 2 weeks) as per the routine immunization schedule [34].

This study revealed wide variances (over 10% difference between the two comparison months and the other months as per the county’s overage coverage) in Pentavalent 3 coverage, Measles/Rubella 1 coverage and the fully immunized children in the two periods. This indicates that there is a large population unreached with the routine facility – based fixed immunization services in the settings. In the implementation of REC strategies, facilities and stakeholders must reconsider the sustainable measures that can complement the routine fixed immunization services in facilities to reach all eligible populations with immunizations. Inconvenient schedules, time constraints between daily socio – economic engagements against seeking immunization services in health facilities have been documented elsewhere as key to seeking immunization services [35]. More emphasis is required during the microplanning for immunization services to ensure that facilities identify their challenges and local solutions including planning for regular outreach programs to reach this needy population with immunization services [28, 36].

This study also showed large differences between the Pentavalent 3 and Measles/Rubella drop – outs between the two periods in the facilities at 13.8 and 24.3% respectively. This is an indication that in this population, there is more of a problem with children completing the vaccination series. This finding is similar to a study conducted in 12 high risk health facilities in Congo [8]. Innovative strategies need to be formulated to minimize missed opportunities for vaccination services. These can include verification of the MCH booklet/records at any opportunity under 5 children present at the facility and integration of immunization services in outpatient departments to minimize drop – outs [31, 37].

Communication and community engagement are key elements of successful vaccine delivery [10, 12, 31, 37–39]. Community health volunteers are often relied upon by health facilities for communicating with the public regarding vaccination services. Evidence has shown that community – based service delivery through community health workers can increase maternal, newborn and child health service including immunization utilization in rural, hard-to-reach areas [32, 37, 40]. Advocacy with local traditional and religious leaders, information sharing with communities and building community mobilization networks with support from community “gatekeepers” may help shed light on the felt needs of the communities and build trust between the community and the immunization program.

These findings raise questions about the effectiveness of the Reaching Every Child (REC) strategy, the key vaccine program implementation strategy, in Migori. REC has been extensively evaluated in rural, urban and social – economic hard – to – reach settings [7–9, 12, 13, 41, 42] and relies upon 5 components: planning and management of resources, reaching target populations, linking services with communities, supportive supervision and monitoring for action, for improved vaccination. [39]. Linking services to communities in the urban and social – economic hard – to – reach settings poses a challenge owing to the migratory nature of the population for survival. In addition, use of data to inform siting of mobile outreaches to reach target populations is a weakness identified in the facilities. The effectiveness of REC hinges on clear demarcation of the community to be served, its catchment population for mobilization, planning and monitoring. Use of facility immunization performance data to identify pockets in the community with unvaccinated/unreached children can inform where to invest the constrained resources to improve immunization coverage and maximize impact.

Our findings also reveal that multiple REC strategies and targeted support to facilities or wards with poor immunization coverages and high drop – outs can sustain the subcounties’ performance above the WHO target 80% [17]. For measles immunization in particular—bearing in mind that near 90% coverage is considered necessary for herd immunity, additional actions including Child Health Days/Weeks, integration of vaccination in other child health activities and supplemental immunization activities and campaigns need to be viable complementary options to improve immunization coverage in these special populations [43].

The authors acknowledge the limitations of this study. These include the small number of link facilities that provided door – to – door services and the short interval for comparing the findings. Use of DHIS2 data is likely to have quality issues (timeliness, accuracy and completeness). However, the project team supported the subcounty teams conduct verifications with individual health facilities before entering in the health information system. The facility EPI targets assigned by the MOH departments are mere estimates based on available demographic data that may not be very accurate hence performance as per the results may be over 100% for some of the antigens.

## Conclusions

Complementing the fixed – point facility immunization with a mobile immunization strategy improved the immunization access and utilization in the health facilities for all antigens. This study stimulates the applicability of some components of the REC approach in the 64 health facilities in Migori particularly the need for novel approaches to planning, calculating coverage, defaulter tracking and social mobilization in rural, urban and economic hard – to – reach environments and monitoring and evaluation of immunization performance using the modern visual applications for decision - making. A mobile immunization strategy underscores the importance of community – driven approaches to improve the access and utilization of immunization services.

## Recommendations

Our findings call for the following key recommendations to improve the immunization services in the county with significant multiple hard to – reach hotspots: 1) Health facilities should actively use routine immunization data to identify hard – to – reach areas to be targeted with outreaches to improve immunization coverage; 2) Outreach (mobile) immunization services should be more flexible to change locations and times of services provision in conformity with the ‘socio – economic’ and migratory patterns of the residents; 3) Counties should invest more resources in strengthening the community health systems so that CHVs are motivated and retained to carry out demand creation, deliver community services, and that communities continue to demand and utilize health services among them immunization; 4) An in – depth qualitative analysis to understand the best strategies and solutions to guarantee a sustained access and utilization of immunization services for the special hard – to – reach populations.

## Data Availability

The datasets used and/or analyzed during the current study are available from the corresponding author on reasonable request. The data was extracted from the Kenya Health Information System (KHIS), formerly the District Health Information System 2 (DHIS2), an open source public access system where all MOH reporting is done. The link to the databases used is https://hiskenya.org/dhis-web-pivot/.

## Abbreviations

CHA: Community Health Assistant
CHV: Community Health Volunteer
CI: Confidence Interval
DHIS: District Health Information System
FIC: Fully Immunized Child
KEPI: Kenya Expanded Programme on Immunization
MCH: Mother and Child Health
MOH: Ministry of Health
QGIS: Quantum Geographic Information System
REC: Reaching Every Child/Community
RED: Reaching Every District
RRI: Rapid Results Initiative
